# Challenges and Progress with Diagnosing Pulmonary Tuberculosis in Low- and Middle-Income Countries

**DOI:** 10.3390/diagnostics8040078

**Published:** 2018-11-23

**Authors:** Anthony D. Harries, Ajay M.V. Kumar

**Affiliations:** 1 International Union Against Tuberculosis and Lung Disease, 68 Boulevard Saint Michel, 75006 Paris, France; akumar@theunion.org; 2 London School of Hygiene and Tropical Medicine, Keppel Street, Bloomsbury, London WC1E 7HT, UK; 3 International Union Against Tuberculosis and Lung Disease, South-East Asia Regional Office, C-6, Qutub Institutional Area, 110016 New Delhi, India

**Keywords:** tuberculosis, World Health Organization, smear microscopy, molecular diagnosis, Xpert MTB/RIF, chest radiography, urine LAM, TB-LAMP, culture and drug susceptibility testing, line probe assays

## Abstract

Case finding and the diagnosis of tuberculosis (TB) are key activities to reach the World Health Organization’s End TB targets by 2030. This paper focuses on the diagnosis of pulmonary TB (PTB) in low- and middle-income countries. Sputum smear microscopy, despite its many limitations, remains the primary diagnostic tool in peripheral health facilities; however, this is being replaced by molecular diagnostic techniques, particularly Xpert MTB/RIF, which allows a bacteriologically confirmed diagnosis of TB along with information about whether or not the organism is resistant to rifampicin within two hours. Other useful diagnostic tools at peripheral facilities include chest radiography, urine lipoarabinomannan (TB-LAM) in HIV-infected patients with advanced immunodeficiency, and the loop-mediated isothermal amplification (TB-LAMP) test which may be superior to smear microscopy. National Reference Laboratories work at a higher level, largely performing culture and phenotypic drug susceptibility testing which is complemented by genotypic methods such as line probe assays for detecting resistance to isoniazid, rifampicin, and second-line drugs. Tuberculin skin testing, interferon gamma release assays, and commercial serological tests are not recommended for the diagnosis of active TB. Linking diagnosis to treatment and care is often poor, and this aspect of TB management needs far more attention than it currently receives.

## 1. Introduction

Tuberculosis (TB), despite being an ancient disease that has affected mankind for thousands of years, remains a huge global public health threat. With TB perceived to be out of control in the early 1990s, the World Health Organization (WHO) took the extraordinary step of declaring TB to be “a global emergency” in 1993, and in 1995 the organization launched the “DOTS (directly observed treatment, short course)” strategy. This evolved into the Stop TB Strategy ten years later and then into the End TB Strategy which started in 2015 [1]. The goal of the End TB Strategy is to end the TB epidemic, the targets for meeting this goal being 90% reduction in TB deaths and 80% reduction in TB incidence by 2030 compared with 2015, with no affected families facing catastrophic financial costs (Table 1). While these successive strategies have resulted in huge progress and millions of deaths averted [2], major challenges still loom large.

In 2016, there were an estimated 10.4 million new patients with TB and 1.7 million deaths, establishing the disease as the ninth leading cause of death worldwide and the leading cause from a single infectious agent, ranking above HIV/AIDS [2]. One of the most critical problems relates to case finding and diagnosis. In 2016, only 6.3 million new patients with TB were reported, which means that 4.1 million patients (40% of the estimated disease burden) were either not diagnosed or were diagnosed but not notified to national programmes or authorities [2]. Drug-resistant TB has also grown inexorably in the last decade, with 600,000 new cases estimated to have resistance to rifampicin (RR-TB), of which 490,000 had resistance to both rifampicin and isoniazid, the two most effective first-line drugs (multidrug-resistant TB—MDR-TB) [2].

Pulmonary tuberculosis (PTB) accounts for the majority of TB disease in both adults and children worldwide. It is from this type of TB that the infection is transmitted to others through droplet nuclei spread from actions such as coughing, sneezing, and talking. The TB control strategies over the years have emphasized the decentralization of TB diagnosis away from central and tertiary hospitals to district hospitals and peripheral health centers to improve patient access and result in earlier diagnosis of the disease.

This paper therefore focuses on progress made and challenges faced with case finding and diagnosis of PTB in low- and middle-income countries (LMIC). It focuses first on district hospitals and peripheral health facilities and then discusses the systems of sending sputum specimens to and the use of national reference laboratories for drug susceptibility testing for first- and second-line drugs. The important challenges in linking patients diagnosed with PTB to care and treatment are discussed within these sections. The paper finally lists the diagnostic tests that should not be used for the diagnosis of active TB disease. In the conclusion, the authors recommend that point-of-care molecular technology is the best way forward for diagnosis if we are to meet the ambitious goal of ending TB by 2030.

## 2. Case Finding and Presumptive TB

In most TB endemic settings, TB case finding is based on a passive or patient-initiated approach—namely, waiting for symptomatic patients to seek health care. The most important clinical features in adults for suspecting the diagnosis of PTB are cough for more than 2 weeks (especially if there is no response to antibiotics), fever, night sweats, and weight loss. In HIV-infected patients, “cough for longer than 2 weeks” as a screening criterion for TB is too insensitive and misses many TB patients [3]. As a result, WHO has revised its guidelines and recommends that any HIV-infected patient with one or more of the following symptoms of cough of any duration, weight loss, fever, or night sweats be regarded as having presumptive TB and be investigated for TB [4].

The strategy of passive case finding has been consistently shown in TB prevalence surveys to miss a large number of TB patients in the community, and active case finding has therefore been proposed as an alternative approach, especially for early diagnosis of TB [5]. Active case finding is essentially a screening intervention initiated by the health services, in contrast to passive case finding which is initiated by symptomatic individuals [6]. Factors influencing the choice of which populations to consider for active case finding include risk and prevalence of TB, accessibility, acceptability, infrastructure, and resources. WHO has provided guidelines for the systematic and active screening of high-risk groups which include household and other close contacts of index patients, people living with HIV, those who have worked in places with silica exposure, those with fibrotic lung lesions on a chest X-ray, prisoners, those living in urban slums, the homeless, and migrants or refugees [6]. The scale and focus of active case finding interventions will inevitably be determined by the local epidemiology of TB and the available resources.

## 3. TB Diagnosis at District Hospitals and Peripheral Health Facilities

There are a number of diagnostic tests for PTB which can be carried out at district hospitals and peripheral health centers (Table 2).

### 3.1. Sputum Smear Microscopy

Sputum smear microscopy has been the mainstay of TB diagnosis for over 100 years, and due to its availability and simplicity, it remains the primary diagnostic technique in many high-burden TB settings. In these countries, most laboratories use smears of unconcentrated sputum (direct smears) with Ziehl–Neelsen staining. In the past, it was recommended that patients with presumptive TB submit three sputum specimens, one of which was an early morning specimen. Due to the extra burden imposed on patients who have to make several journeys to the health facility, the laboratory technicians who have to examine three smears for each patient, and evidence that two sputum specimens are as sensitive as three, WHO now recommends that two sputum specimens of good quality collected at any time of the day (spot specimens) are sufficient [7].

Sputum smear microscopy, unfortunately, is a relatively insensitive test. A concentration of 10,000 bacilli per mL of sputum is generally required for a smear result to be positive. Lack of pulmonary cavitation in people living with HIV and the resulting low bacillary concentrations in sputum mean that smear microscopy is often negative in more than half of patients with HIV-associated TB [8]. Sensitivity for the same reasons is also reduced in children and those with extra-pulmonary disease. Using fluorescence microscopy and modifying sputum processing methods, for example, by using bleach, can improve sensitivity [9], but these techniques can be complex, have cost implications, and are not always popular with busy laboratory technicians for whom smear microscopy is just one of many tasks needing to be performed. The other shortcomings of smear microscopy include the inability of the test to distinguish between viable and nonviable organisms, between *Mycobacterium tuberculosis* (*MTB*) and non-tuberculous mycobacteria, and, finally, between drug-susceptible and drug-resistant strains of the organism. For these reasons, WHO recommends that TB Programmes transition to replacing microscopy as the initial diagnostic test with approved rapid diagnostics that allow for the simultaneous detection of *MTB* and drug-resistant TB [7].

### 3.2. Xpert MTB/RIF

One of the “game changers” in the diagnosis of TB in the last decade has been the introduction, deployment, and scale-up of the Xpert MTB/RIF assay (Cepheid Inc, Sunnyvale, CA, USA). This is a highly sensitive and specific, fully automated, and commercially available nucleic acid amplification test for use with sputum and other body specimens [10]. The assay uses real-time polymerase chain reaction technology to detect *MTB* and the specific rifampicin resistance mutation *rpoB*. The cartridge-based system avoids the need for prior sputum processing, requires minimal expertise, and has a short sample processing time of two hours to confirm *MTB* and detect rifampicin resistance.

Multi-country evaluations have confirmed the operational feasibility, accuracy, and effectiveness of Xpert MTB/RIF at district and subdistrict health facilities in TB-endemic countries in Africa, Asia, and Latin America [10,11], persuading WHO to support the wider use of this technology. WHO initially recommended Xpert MTB/RIF for those suspected of having MDR-TB and for those with presumptive TB who were also co-infected with HIV [12]. However, in 2013, WHO made a conditional recommendation that Xpert MTB/RIF be considered as the initial diagnostic test for all people requiring investigation for TB [13], and more recently advised that programmes transition away from smear microscopy and prioritize the initial use of Xpert MTB/RIF [7]. The sensitivity and specificity of Xpert MTB/RIF for the diagnosis of PTB in adults are approximately 90% and 98%, respectively, using culture as the reference standard [14]. In a multicenter study, sensitivity was better in HIV-negative compared with HIV-positive patients: in HIV-negative patients, sensitivity was 99.0% in smear-positive and 77.5% in smear-negative patients, and in HIV-positive patients, sensitivity was 97.7% in smear-positive and 71.8% in smear-negative patients [11]. In children sensitivity is lower at between 65% and 76%, with specificity between 99% and 100%, but the assay does help to increase the proportion of children with a laboratory-confirmed diagnosis of TB [14].

Between 2010 and 2016, nearly 6700 Xpert MTB/RIF instruments were procured by National TB Programmes in 130 of the 145 countries that were eligible for concessional pricing, and in 2016 alone this included the procurement of nearly 7 million test cartridges [2]. These impressive statistics, however, belie major challenges that threaten the efficient functionality of Xpert MTB/RIF such as the need for stable and regular electricity, fully functional instrument modules, adequate maintenance, basic computer training, and uninterrupted cartridge supplies [15,16]. Poor utilization of the instruments is also a problem, especially as countries start scaling up and using the new technology [17,18,19].

The basic premise of Xpert MTB/RIF is improved patient access to early and accurate diagnosis which, in turn, should decrease morbidity and mortality associated with diagnostic delay, losses to follow-up, and mistreatment. Two randomized trials showed that Xpert MTB/RIF at the primary care level was associated with a shortened time to diagnosis and treatment, and especially so in those with associated HIV infection [20,21]. However, in one of these trials and in another study which was part of national roll-out of Xpert technology in South Africa, the molecular diagnostic assay did not reduce mortality at 6 months compared with routine diagnostic smear microscopy [20,22]. These disappointing and rather unexpected negative findings of the trials related to higher rates of empiric TB treatment in the microscopy compared with the Xpert arms along with intrinsic health system weaknesses [23]. 

Despite these failures so far to improve treatment outcomes, the field of molecular technology for TB diagnosis is dynamic and fast developing. The Xpert OMNI system is a portable, battery-operated, single-cartridge system which is due to be launched in 2019 and would in principle allow the decentralization of diagnosis to the primary health care level and community [24]. The Xpert MTB/RIF Ultra is an assay with higher sensitivity than Xpert MTB/RIF, is run on the same instrument as Xpert, and requires only a software upgrade [25]. In 2017, WHO recommended that Xpert MTB/RIF Ultra replace the first-generation Xpert MTB/RIF [26]. Studies at the field level, however, need to be conducted to assess what programmatic advantages this new assay has over the first-generation assay.

Finally, there is a new automated, cartridge-based assay that has been developed which accurately detects *MTB* mutations associated with resistance to isoniazid, fluoroquinolones, and aminoglycosides [27]. Current Xpert MTB/RIF assays cannot detect isoniazid resistance, with rifampicin resistance being taken as a proxy for the diagnosis and treatment of MDR-TB. However, there is evidence that rifampicin-susceptible, isoniazid-resistant TB, which occurs in 8% of cases globally [28], is associated with poorer treatment outcomes when treated with a standard first-line regimen [29], and WHO recommends a change in first-line TB treatment to compensate for this [28]. WHO has also recently recommended a new short course regimen of 9–12 months for MDR-TB, provided patients have not been treated with second-line drugs and/or have had resistance to fluoroquinolones and second-line injectable agents excluded [30]. Currently, conventional mycobacterial culture and second-line drug susceptibility testing or molecular line probe assays performed in national reference laboratories are the only ways of obtaining this information, but the new molecular technology assay described earlier may change this in the future and allow these diagnoses to be made at more peripheral levels of the health system.

### 3.3. Chest Radiography

Chest radiography, including the latest advances such as digital radiography and computer-assisted radiography, is a useful complementary tool for the diagnosis of clinical TB. In general, patients with positive sputum smears or positive Xpert MTB/RIF results do not need a chest X-ray unless there are symptoms or signs such as dyspnoea to suggest additional complications with pneumothorax, pleural effusion, or pericardial effusion. Patients with presumptive TB in whom smears and Xpert MTB/RIF results are negative should be reviewed by the treating clinician and a decision made about treating for clinical TB or not. This decision is often dependent on the findings of the chest radiograph. Unfortunately, no chest radiographic pattern is absolutely diagnostic of TB, although upper lobe involvement, cavitation, fibrosis, and bilateral disease are suggestive of TB in adults. One approach is to treat all presumptive TB patients with negative bacteriological confirmation of TB and abnormal chest radiographs with a course of broad-spectrum antibiotics. Those with persistent symptoms should be reviewed, repeat sputum specimens collected for examination, and a decision made to treat or not for clinical pulmonary tuberculosis.

Patients with HIV-associated TB cause particular difficulty. In the immunosuppressed patient, as the CD4 cell count declines, the chest radiograph becomes increasingly atypical. Over 20% of patients with smear-negative, culture-positive PTB may have a completely normal chest X-ray [31]. In these circumstances, it is easy to miss the diagnosis of TB completely and manage the patient as though he/she has the HIV wasting syndrome. These sorts of diagnostic mistakes in resource-poor settings are associated with high mortality. A systematic review of autopsy studies in Africa and Asia amongst patients dying from AIDS found that 40% or more had TB, much of which was not recognized or diagnosed in life [32].

### 3.4. Urine LAM

A useful TB diagnostic test for HIV-infected patients with advanced immunodeficiency is measurement of urine lipoarabinomannan (LAM), one of the cell wall lipopolysaccharide components of *MTB.* This can be measured either with an ELISA or more easily with a Determine TB-LAM test strip (Alere, Waltham, MA, USA), the latter costing $3.50 per test strip and producing a result in 30 min by the bedside [33]. In HIV-infected patients, specificity is high at over 95% for all CD4 strata. Useful sensitivity is observed in those with CD4 counts of <200 cells/µL, and this progressively increases as the CD4 count decreases. In one study, the sensitivity was 39.0% at CD4 counts of <200 cells/µL, 51.7% at CD4 counts of <100 cells/µL, and 66.7% at CD4 counts of <50 cells/µL; specificity was greater than 98% for all CD4 strata [34]. In HIV-TB patients with advanced immunodeficiency, there is often haematogenous TB dissemination to the kidneys and elsewhere, and this is thought to be the primary mechanism by which LAM enters the urine and results in a positive test [35].

The use of the bedside urine LAM, with a positive result guiding the start of antituberculosis treatment, in HIV-infected adults admitted to hospital in several African countries has been associated with a decrease in overall mortality at 2 months [36]. This is encouraging news, added to which there is evidence that Xpert MTB/RIF can also be used on urine specimens to identify HIV-infected hospitalized adults with TB [37]. A new approach in Malawi and South Africa showed that amongst HIV-infected patients admitted to hospital, a screening package of urine LAM and urine Xpert MTB/RIF in addition to sputum Xpert MTB/RIF resulted in an increase in TB diagnosis and treatment and a reduced overall mortality in key subgroups [38]. Sick HIV-infected patients with pulmonary and disseminated TB often cannot cough up sputum, and the use of urine as a suitable and easy-to-collect specimen for investigation is a novel step forward.

### 3.5. TB-LAMP

Loop-mediated isothermal amplification (LAMP) is a unique, isothermal technique for amplifying DNA that is simple to use with an easy-to-read visual display. TB-LAMP is a manual assay that takes less than one hour to perform and can be read with the naked eye under ultraviolet light. It is therefore suitable for use at peripheral health centers where smear microscopy is performed. In 2016, WHO recommended that TB-LAMP may be used as a replacement for sputum smear microscopy for the diagnosis of pulmonary TB in adults with signs and symptoms of presumptive TB [39]; this was a conditional recommendation with very-low-quality evidence. It can also be considered as a follow-on test to microscopy in adults with signs and symptoms of pulmonary TB, especially when further testing of sputum smear-negative specimens is necessary. The test, however, does not detect drug resistance and is therefore only really suitable for the testing of patients at low risk of drug-resistant TB. 

Experience in Africa at the peripheral health facility level suggests that TB-LAMP can perform better than smear microscopy and is associated with shortened waiting times for diagnosis [40,41]. However, these encouraging findings are not uniform. In a peripheral laboratory in a high-burden setting in Vietnam, results with TB-LAMP were less promising with the data not supporting the use of this test as a replacement for smear microscopy [42]. The jury is therefore still out with respect to TB-LAMP, and further observational studies are required.

### 3.6. Diagnosing TB in Children

The diagnosis of TB in children is a major challenge, mainly because young children are unable to expectorate sputum, and bacteriological confirmation is therefore rarely achieved [43,44]. Most children with pulmonary TB will have a cough (although usually not strong enough to produce a good-quality sputum specimen), and constitutional symptoms often include failure to thrive and reduced playfulness. Clinical signs are unfortunately subtle, and despite attempts to develop diagnostic scoring systems, none have been adequately validated. In clinical practice, chest radiography is one of the most useful diagnostic tests, the finding of hilar lymphadenopathy with or without airway compression being highly suggestive of pulmonary TB [43]. If sputum can be expectorated, this can be examined by smear microscopy and/or Xpert MTB/RIF as described earlier. If sputum cannot be expectorated, semi-invasive techniques can be used such as gastric aspiration, nasopharyngeal aspiration, or sputum induction, the latter showing reasonable sensitivity and high specificity when tested with Xpert MTB/RIF [45]. An alternative method of obtaining sputum is to use a “string test” in which a gelatin capsule containing a nylon string is swallowed and later retrieved for bacteriological examination. The procedure is tolerable for children and the yield of TB is similar to that obtained through sputum induction [46]. Detecting the organism in stool may be another option where Xpert MTB/RIF testing is available, and this approach has been used successfully in HIV-infected and non-infected children in Zimbabwe and Kenya [47,48]. Skilled staff and adequate resources need to be available for these alternative ways of obtaining specimens for bacteriological examination, and as this is not usually the case in low-income countries, a clinical diagnosis of childhood TB is often the norm.

### 3.7. From Case Finding to Diagnosis to Treatment

To identify, diagnose, and treat TB at the district hospital or peripheral health facility, the systems have to work and connect with each other. While in principle this should be straightforward, the reality is very different. A recent review of primary care clinics in South Africa showed that 20% of patients with classic TB symptoms were never screened by health care staff, and 80% of patients who were screened and identified with TB symptoms failed to submit the necessary sputum specimens for investigation [49]. These losses prior to diagnosis are termed “pre-diagnostic loss to follow-up”.

In countries in sub-Saharan Africa, Asia, and the Western Pacific, a systematic review showed that between 4% and 38% of patients diagnosed in the laboratory with sputum smear-positive or culture-positive TB failed to start treatment [50]. This “pre-treatment loss to follow-up” is no better with the use of Xpert MTB/RIF, with 21%–45% of patients with Xpert-confirmed TB failing to start treatment in Cameroon, Myanmar, and South Africa [51,52,53]. For those patients who do get treated, the time taken from identification of a positive specimen to treatment initiation can also be long, and this compromises individual care and increases the risk of transmission of *MTB* infection within families and communities. Much more attention is needed at the programmatic level to link the process of screening to diagnosis and of diagnosis to treatment and to monitor and regularly evaluate this aspect of TB control.

## 4. TB Diagnosis at National Reference Centers

### 4.1. Mycobacterial Culture and Drug Susceptibility Testing

Mycobacterial culture and drug susceptibility testing (CDST) in low- and middle-income countries is usually performed at National Reference Laboratories or in Tertiary Care Hospitals. The usual culture medium is Lowenstein Jensen (with the average time to growth taking between 4 and 6 weeks), although newer and faster liquid culture systems (such as the Mycobacterium Growth Indicator Tube—MGIT) are increasingly being used (with the average time to growth taking between 1 and 2 weeks). Pure growths of *MTB* are usually subcultured and drug susceptibility testing (DST) is carried out using the proportion method [54,55]. CDST is rarely used for individual diagnosis in resource-poor settings because of the long time it takes for *MTB* to grow on culture media. Mycobacterial CDST facilities are more often used for monitoring drug susceptibility patterns in patients with relapse or recurrent tuberculosis, assessing treatment response among drug-resistant TB patients, and monitoring the prevalence of MDR-TB in the community.

### 4.2. Line Probe Assays (LPA)

Line Probe Assays (LPA) are molecular methods based on nucleic acid amplification and the detection of gene mutations. They were initially used to diagnose both isoniazid and rifampicin resistance directly from sputum specimens which were smear-positive for acid-fast bacilli or from positive cultures of *MTB.* LPAs are more rapid than conventional phenotypic DST methods, allowing a diagnosis of MDR-TB within 3 days from either sputum submission or from obtaining a pure culture of *MTB*. Good-functioning and well-resourced laboratories plus well-trained and skilled laboratory technicians are necessary for LPAs to perform well. Various factors determine LPA performance in the laboratory, including the volume of sputum specimens, strict attention to manufacturer’s instructions about process, timing, sequencing, and temperature control [56]. Laboratories that have a high throughput of tests are likely to perform much better than those whose burden of work is small.

In 2016, WHO recommended in patients with a sputum smear-positive specimen or a cultured isolate of *MTB* that a commercial molecular LPA be used as the initial laboratory test instead of phenotypic DST to detect resistance to rifampicin and isoniazid [57]. Once the diagnosis of rifampicin-resistant TB or MDR-TB is made, second-line LPAs can then be used to detect added resistance to second-line drugs such as fluoroquinolones or injectable drugs such as amikacin and kanamycin. It is important to know whether there is extensively drug-resistant TB (XDR-TB—MDR-TB with added resistance to both fluoroquinolones and second-line injectable drugs) or pre-XDR-TB with added resistance to either fluoroquinolones or second-line injectable drugs. Again in 2016, WHO recommended that for patients with confirmed rifampicin-resistant TB or MDR-TB, second-line LPAs be used as the initial test instead of phenotypic culture-based DST to detect resistance to fluoroquinolones and second-line injectable drugs [58].

### 4.3. Rapid Whole-Genome Sequencing

Twenty years have now passed since the publication of the complete genome sequence of *MTB* [59]. Since then, whole-genome sequencing (WGS), which provides a rapid and comprehensive view of the organism’s genotype, has been developed to predict the drug susceptibility status of *MTB,* investigate outbreaks of TB, and to understand transmission of infection within populations [60]. Despite the perceived benefits of WGS for routine diagnosis and management of drug-resistant TB, it has only been implemented in a few high-income countries such as the United Kingdom [61]. Current challenges with this technology include high costs, the technical skill required, the complexity of the data and its analysis, and the unavailability of sequencing facilities. There are currently no plans for the routine implementation of WGS in low- or middle-income countries with a high burden of TB.

### 4.4. Getting Sputum Specimens to National Reference Laboratories

National Reference Laboratories form an important part of the laboratory network within a country, but it is crucial that there are good systems to ensure that the appropriate sputum specimens from district and peripheral health facilities can arrive in a safe and timely way and, equally, that results can be relayed back to health care staff and patients. So often, however, this system fails with sputum specimens from patients with retreatment TB, for example, not being collected or delivered to the national reference laboratories, or when delivery does take place with unacceptable time delays that compromise the chances of obtaining pure cultures of *MTB* that are needed for phenotypic DST or LPA [62,63,64,65]. Reliable and efficient transport and monitoring systems need to be put in place, but these are costly, difficult to maintain, and are often not accorded the priority they deserve.

## 5. Tests That Should Not Be Used for the Diagnosis of Active TB

### 5.1. Tests for Diagnosing Latent TB

There are two tests available for diagnosing latent TB infection: the tuberculin skin test (TST) and the interferon gamma release assay (IGRA) [66]. The TST consists of an intradermal injection of 5 tuberculin units of purified protein derivative (PPD-S) or two tuberculin units of PPD RT23, with the response read 48–72 h later, requiring the patient to revisit the clinic after the injection has been administered. Challenges in its use include false positive results from previous Bacillus Calmette-Guerin (BCG) vaccination or from non-tuberculous mycobacteria and false negative results due to malnutrition and HIV-related immunosuppression. The IGRA is an in vitro blood test of the cell-mediated immune response following interferon gamma stimulation of one or more specific antigens to *MTB.* IGRA is therefore more specific than TST, particularly in people who have received BCG, and the test only requires one visit by the patient. However, a blood sample is required, there needs to be a laboratory with appropriately trained staff, and the test is costly.

Both tests are unable to distinguish between latent TB infection and active TB disease and are also poor at predicting who with latent TB infection will progress to active disease. They are therefore not used for the diagnosis of active TB, except for TST which may form part of the assessment package used in diagnosing childhood TB (typical clinical features, family history of contact with an index case, abnormal chest radiography, and positive TST).

### 5.2. Serological Tests

Diagnostic tests for active TB based on the detection of antibodies (serological tests) have been commercially available for decades, although international guidelines do not recommend their use. Findings from a systematic review and meta-analyses of the accuracy of serological antibody and antigen tests for the diagnosis of PTB suggest that these tests are inaccurate and imprecise [67,68]. In 2011, the WHO issued a strong recommendation against the use of all commercial serological tests for the diagnosis of TB disease [69], and this recommendation still holds to this day.

## 6. Ways Forward and Conclusions

In 2015, in order to accelerate the ending of the TB epidemic, the Stop TB Partnership launched the 90-(90)-90 TB diagnostic and treatment targets (Table 3) [70]. Finding and diagnosing patients with TB is essential to meeting these targets. While smear microscopy remains the primary diagnostic test at the district and peripheral facility level, molecular technology is gradually taking over because of the ease of testing and the increasing need to know the drug susceptibility pattern of *MTB* before starting treatment.

Currently, the use of Xpert MTB/RIF is plagued by health system and infrastructure challenges, but these can be resolved if there is the political will matched with adequate resources. The urgent need moving forward is to further develop Xpert MTB/RIF to be a point-of-care diagnostic test that has good sensitivity for identifying *MTB* and drug susceptibility patterns, especially for isoniazid, rifampicin, fluoroquinolones, and second-line injectable agents. The battery-operated Xpert OMNI system has the potential to allow molecular technology to be decentralized to peripheral health facilities, but the instrument and the assay have to be at an affordable price for this dream to be realized. The molecular technology platforms must advance to take on testing for isoniazid and second-line drugs so that treatment can be rapidly adjusted to the drug susceptibility status of the patient. The combinations of urine LAM with sputum and urine Xpert MTB/RIF in HIV-infected adults [38] and of urine LAM with stool Xpert MTB/RIF in HIV-infected children [47] are powerful tools for diagnosing pulmonary TB in these HIV-infected populations, and these should be scaled up at the programmatic level in high HIV–TB burden countries. The diagnosis of pulmonary TB in children remains a challenge, but it is encouraging to see that this is a top research priority with international calls for the development of a rapid point-of-care diagnostic test that uses easy-to-obtain samples and markers of the host’s immune response to TB (as opposed to trying to identify *MTB* itself) [71,72].

Better and more rapid diagnosis in adults and children must be linked to quicker initiation of treatment. For this to happen there needs to be far better communication between diagnostic laboratories and treatment units, and a key component of supervisory activities from central and regional units of TB Control Programmes must be to check that all patients with bacteriologically confirmed TB have started treatment [73]. One further action that might improve the current situation is for the WHO and national TB Control Programmes to insist on using diagnosed patients as the denominator for assessing treatment outcomes rather than the patients registered for treatment [73].

The conclusion of the first ever United Nations General Assembly High-Level Meeting on TB (UNGA-HLM) on 26 September 2018 gives grounds for optimism. The declarations of member states and implementation of the pledges made will all come under the remit of the WHO which, in turn, needs to fully engage and work together with all relevant stakeholders, including governments and civil society [74]. UNGA-HLM should quickly yield increased and sustained financing that can allow key deliverables to be met and the tide to be turned on this global epidemic.

## Figures and Tables

**Table 1:** The WHO milestones and targets for ending tuberculosis by 2030.

Indicators	Interim Milestones		Targets
	2020	2025	2030
Percentage reduction in absolute numbers of TB deaths (compared with 2015 baseline)	35%	75%	90%
Percentage reduction in TB incidence rate (compared with 2015 baseline)	20%	50%	80%
Percentage of TB-affected households who experience catastrophic costs due to TB (level in 2015 unknown)	0%	0%	0%

TB = tuberculosis. Adapted from [1].

**Table 2:** TB diagnostic tests for use at district hospitals and peripheral health facilities.

Diagnostic Test	Advantages	Disadvantages
Sputum smear microscopy for AFB	Long experience, inexpensive, and uses light microscopy	Cumbersome for laboratory staff and patients
	Identifies the most infectious patients	Does not identify drug resistance
	Used for follow-up of patients on treatment	Does not distinguish viable and nonviable AFB
		Does not distinguish *MTB* from non-tuberculous mycobacteria
Xpert MTB/RIF	Easy to use and rapid results in 2 h	Instruments and cartridges expensive
	Identifies rifampicin resistance	High infrastructure and maintenance needs
Chest radiograph	Long experience with use	No radiographic pattern absolutely diagnostic of TB
		Atypical and normal radiographs with HIV advanced disease
Urine LAM	Inexpensive and easy to use at the bed side with test strip	For use in HIV-infected patients
	Rapid results in 30 min	Sensitivity increases as CD4 cell counts decrease
TB-LAMP	Simple to use, easy visual display and results in one hour	Does not identify drug resistance

TB = tuberculosis; AFB = acid-fast bacilli; *MTB* = *Mycobacterium tuberculosis*; RIF = rifampicin; LAM = lipoarabinomannan; LAMP = loop-mediated isothermal amplification.

**Table 3:** 90-(90)-90 stop TB partnership global targets for tuberculosis.

Reach at least 90% of all people with TB ^a^ As a part of this approach, reach at least (90%) of the key populations ^b^ Achieve at least 90% treatment success for all people diagnosed with TB ^c^

TB = tuberculosis. ^a^ includes people with both drug-susceptible and drug-resistant TB; ^b^ includes vulnerable, underserved, and at-risk populations which vary depending on country context; ^c^ includes achieving 90% treatment success among people diagnosed with both drug-susceptible and drug-resistant TB. Adapted from [70].
